# Exploring the efficacy of low-level laser therapy and exercise for knee osteoarthritis

**DOI:** 10.17159/2078-516X/2019/v31i1a6058

**Published:** 2019-01-01

**Authors:** A Kholvadia, D Constantinou, P J-L Gradidge

**Affiliations:** Centre for Exercise Science and Sports Medicine, Faculty of Health Sciences, University of the Witwatersrand, Johannesburg, South Africa

**Keywords:** photobiomodulation, physical therapy, degenerative joint disease

## Abstract

**Background:**

Knee Osteoarthritis (KOA) is a prevalent, chronic disorder with excessive functional, social and economic burdens. The goal of treatment is to alleviate the symptoms and slow the progression. Documenting the effects of exercise and LLLT as co-modalities in the management of KOA allows practitioners to implement this management tool as part of KOA rehabilitation, resulting in the earlier discharge from a supervised rehabilitation setting.

**Objective:**

The purpose of this study was to determine the effect of low-level laser therapy (LLLT) in the treatment of knee osteoarthritis (KOA). A randomised controlled trial (RCT) was conducted on 111 participants (aged between 40–75 years) diagnosed with KOA. Participants were randomised into an exercise (n=39), LLLT (n=40), or a combined exercise-LLLT (n=32) group.

**Methods:**

The Western Ontario and McMaster Universities Osteoarthritis Index (WOMAC) scale was used to assess pain and functionality. Knee range of motion was assessed using a goniometer, and the one-minute timed sit–to-stand test measured physical functionality at four time points: (T1) baseline, (T2) post 12-session intervention, (T3) one-month post intervention and (T4) three-month’s post intervention. Knee circumference was measured using a measuring tape.

**Results:**

WOMAC pain and functionality scale and knee circumference scores decreased in all three groups (P<0.05), but the combined exercise-LLLT group demonstrated better outcomes than the LLLT or exercise alone groups respectively. The combined exercise-LLLT group showed better acute and long-term benefits with participants experiencing a 3.5 centimetre decrease in knee circumference, 24 point improvement in the WOMAC pain and functionality scale, and a four repetition increase in physical functionality.

**Conclusion:**

The findings suggest that LLLT is a viable tool for managing KOA when used in conjunction with physical exercise.

Knee osteoarthritis (KOA) is a prevalent, chronic disorder with functional, social and economic burdens.^[1]^ The World Health Organization’s (WHO) Global Burden of Disease 2010 study highlighted that, with Africa’s attention on infectious disease, child and maternal health, the burden of non-communicable diseases, such as KOA, has amplified.^[2]^ A prevalence of 33% has been observed amongst older rural South Africans, whilst South Africans in urban areas show a higher prevalence of KOA (55%).^[3]^ Urbanisation has resulted in increased life expectancy, increased ageing populations and higher levels of obesity-making osteoarthritis the fourth leading cause of disability by the year 2020.^[2]^ Furthermore, common KOA-related symptoms include pain and joint stiffness which negatively influences disability and functionality.^[1,2]^

Contemporary evidence for identifying, diagnosing and managing KOA varies, depending on the stage of disease progression and severity.^[1]^ The main goal of management for KOA is to alleviate the symptoms and delay the progression of the disease,^[4]^ with therapeutic modalities, including physical therapy, pharmacological intervention, and surgery.^[4]^ The current treatment regimen for KOA aims at controlling pain and improving the joint function to enhance functionality through pharmacological intervention and physical therapies.^[4]^ With disease progression, surgical intervention is considered as activities of daily living decreases.^[4]^

Physical rehabilitation is important as a tool for disease management in the early phases of KOA diagnosis, particularly as evidence shows that compared with sedentary patients, those patients who underwent exercise therapy had significantly better outcomes.^[5]^ Contemporary evidence demonstrates that low-level laser therapy (LLLT) (also known as photobiomodulation) in particular, together with physical therapy, has the potential for better outcomes than conservative therapy alone.^[6,7,8]^ Data from these studies observed that participants in the group using LLLT and combined with exercise experienced increased pain relief and improved joint functionality and mobility. However, there are other studies showing no pain relief with LLLT alone.^[9,10]^ Given the conflicting outcomes from these studies, the aim of this study was to investigate the efficacy of combined LLLT and exercise compared with exercise or LLLT alone in a South African cohort of patients diagnosed with KOA.

## Methods

### Study design

A descriptive, intervention study design was used to evaluate the differences between the groups participating in LLLT and exercise alone, and a combination of the two modalities. Participants were randomised into one of the three groups using pre-sealed envelopes attached to the batch of documents provided to prospective participants at baseline testing. A single blind methodology was employed as the principal investigator carried out the intervention programmes. The study participants were unaware of the diverse intervention modes of the study. Furthermore, a factorial design experiment, which is a variation of the between-group design experiment, was used. This study consisted of three treatment variables which examined the independent and simultaneous effect of the respective treatments on the outcome measures of the knee’s range of motion (ROM), knee circumference, WOMAC pain scale, and physical functionality. This research design allowed the study to explore the effects of each treatment separately, together with the effect on the variables explored, thereby providing a rich and encompassing multidimensional view. The data collection for the randomised controlled trial (RCT) was conducted at a biokinetics rehabilitation centre in Johannesburg, South Africa.

### Participants

Male and female knee osteoarthritis patients (n=120) from Johannesburg, aged 40–75 years, and meeting all the study’s inclusion criteria, were recruited to voluntarily participate in this randomised control trial after being referred to the study by various medical practitioners. Prospective participants were excluded from the study if they were pregnant, diagnosed with cancer or epilepsy and if they were physically unable to complete one or more tests in the battery of physical tests required for the study. The participants were randomly allocated to an exercise group, a LLLT group or a combination of exercise plus LLLT group respectively. Sealed envelopes were used and attached to the initial testing documentation after the baseline testing (T1) was completed. These pre-sealed envelopes were marked and randomly attached to the back of each batch of baseline testing forms indicating an assignment to a prospective intervention group. There was a dropout rate of 0%, 2.5% and 20% in the exercise, LLLT and combination treatment groups respectively. One hundred and eleven participants completed the study (exercise group, n=39, LLLT group, n=40 and the combined exercise-LLLT group, n=32).

All participants were thoroughly informed regarding consent to participation and withdrawal, testing procedures and intervention programmes. Written informed consent was obtained from all participants prior to data collection (T1). Ethics approval was obtained from the Faculty of Health Sciences Human Research Ethics Committee (Medical) at the University of the Witwatersrand (certificate number: M161112).

### Knee circumference

Knee circumference was assessed using a measuring tape while the participant was supine; with the affected knee supported by a towel to create a 30° flexion in the knee which allowed the relaxation of the quadriceps muscle. ^[11]^ The site of joint circumference measurement was proximal (two cm above the mid patella), mid and distal (two cm below the mid patella) recorded to the nearest millimetre (mm).

### Pain and functionality management

The Western Ontario and McMaster Universities Arthritis Index (WOMAC) is a self-administered questionnaire which can be used to describe and evaluate pain and function in patients with osteoarthritis of the knee. This questionnaire was chosen for its validity and reliability as a tool in reporting KOA.^[12]^ The WOMAC measures five items for pain (score range 0–20), two for stiffness (score range 0–8), and 17 for functional limitation (score range 0–68). Questions regarding physical functioning cover everyday activities such as stair use, standing up from a seated or lying position, bending, walking, getting in and out of a car, shopping, putting on or taking off socks, lying in bed, getting in or out of a bath, sitting, and doing heavy and light household duties. ^[12]^ The change in the WOMAC score was used as the outcome variable of pain and functionality during the study.

### Knee range of motion (ROM)

Bilateral ROM was measured using a Baseline® goniometer to the nearest degree (°) while the participant was supine.^[11]^ For flexion measurements the participant was asked to bring the foot as close to the buttocks as possible. For extension measurements, the participant was instructed to actively straighten the knee.

### Physical functionality

The one-minute timed sit-to-stand test was used to measure physical functionality.^[13]^ The participant was requested to sit in the middle of a 50 cm high chair with his/her arms crossed against the chest, feet flat on the floor and back straight up against the backrest of the chair. The participant was then instructed to sit and stand respectively. A stopwatch was used to count down 60 seconds and the number of completed repetitions recorded.

### Intervention

There were three intervention groups, i.e. exercise, LLLT, and a combined exercise-LLLT.

#### Exercise group

The exercise programme was conducted three times per week and consisted of 12 sessions based on rehabilitation guidelines.^[5]^ The exercise protocol included four different types of exercises, i.e. flexibility (quadriceps, calf muscles and hamstrings), stability (quad setting, single leg balance), strength and endurance (supine and prone straight leg raises, abductor squeezes, step-ups, calf raises), designed to maintain and improve knee functionality through improved muscular strength, ROM and locomotor function of the knee joint. The programme was self-paced, starting at a low intensity and became progressively more challenging. The principal investigator supervised all exercise sessions and determined individual participant exercise progression.

#### Low-level laser therapy (LLLT) group

Participants in the LLLT group were exposed to three different arrays as part of the LLLT protocol over a period of 12 sessions, with each session progressing from 35 min to 45 min.^[14]^ There are no frequency protocol guidelines on the use of LLLT for KOA, therefore sessions were scheduled for times a week to maintain uniformity across all three intervention groups. A circumferential application method was employed. Three placements with medial and lateral applications overlapping at the patella’s surface were used. The participant’s knee was set at 60°–70° for optimal penetration of light from the light-emitting diodes (LEDs).^[14]^

#### Combined exercise and LLLT group

The combined exercise and LLLT group underwent a protocol that included both the exercise rehabilitation and the LLLT intervention programmes described above. The exercise sessions were done preceding the LLLT intervention. These sessions were scheduled for three times a week.

### Statistical analysis

Statistical analysis was performed using Statistica v13.3 (TIBCO Software. StatSoft Inc.). After assessing the normal distribution of the data using the Shapiro-Wilks test, descriptive data were presented as mean ± standard deviation (SD). The differences between baseline and post-study period analysis of covariance (ANCOVA) are presented as effect sizes (Cohen’s d) and the differences between study groups were determined using ANOVA (analysis of variance). Paired t-tests were used to measure differences between baseline and post-intervention data for all variables. Independent t-tests were performed to determine if the intervention groups differed in basic participant characteristics. Repeated measures ANOVA tests with between-subjects effects (exercise vs. LLLT or exercise vs. combination group) and within-group effects (T2, T3, T4) were performed to indicate the differences in measurements from baseline. Significance was accepted at p<0.05.

## Results

### Participant characteristics

The one hundred and eleven participants in the study were randomly allocated into the three intervention groups exercise (n=39), LLLT (n=40), and combined exercise-LLLT (n=32) groups, respectively. Most of the participants were women (n=85, 77%), with a mean age of 61.8 ± 5 years. The exercise group exhibited the highest BMI at 36.1 ± 3.7 kg/m^−2^) followed by the LLLT group (31.6 ± 4.5 kg/m^−2^) and the combined exercise-LLLT group (29.6 ± 4.7 kg/m^−2^).

### Knee osteoarthritis variable outcomes

#### Knee circumference

Varying effects were observed in all three groups for the key outcomes. However, the combined exercise-LLLT group demonstrated improved efficacy for the outcome measures of knee circumference While the post-intervention (12-week) knee circumference decreased significantly in all groups (p<0.05), this effect was highest in the LLLT group compared to the combined exercise-LLLT and exercise groups (Table 2).

#### WOMAC pain and functionality scale

Table 2 shows that all groups experienced improvements in the WOMAC pain and functionality scale, but this was most noticeable in the combined LLLT-exercise group (p<0.05). The values at baseline were comparative in all groups. However, the exercise vs. LLLT had significantly greater effect sizes at T3 and T4 compared to the exercise and combined exercise-LLLT groups.

#### Knee ROM

Knee extension ROM was significantly lower in the combined exercise-LLLT and LLLT groups compared with the exercise group at baseline (Table 1). Only the combination group experienced significant improvements following the intervention (Table 2). Baseline knee flexion ROM was higher in exercise group compared with the LLLT and combined exercise-LLLT group (p<0.005); however, the effect of the intervention was only significant in the combined exercise-LLLT group (Table 2).

#### Sit-to-stand

Baseline values for physical functionality (measured by the sit-to-stand test) were similar across all three intervention groups. Table 2 demonstrates that the combined exercise-LLLT group had the greatest improvements in the sit-to-stand repetitions following the intervention, and this effect was evident at the T3 and T4 time points.

## Discussion

This study aimed to evaluate the effect of utilising LLLT in combination with exercise in comparison to the use of exercise or LLLT as stand-alone therapies for the conservative management of patients diagnosed with KOA. Varying effects were observed in all groups for the key KOA study outcomes; however, the combined exercise-LLLT group demonstrated the best effects when compared with the exercise or LLLT groups alone.

The decrease in knee circumference values and improvements in the WOMAC pain and functionality scores indicate structural enhancement in the joint. A decrease in joint effusion was evident by the decrease in knee circumference scores and an improvement in WOMAC pain and functionality score, as supported by other authors such as Fukuda et al. ^[7]^, Alfredo et al.^[8,6]^. Improvements of between 11 and 12.9 points in a period of 2–6 months post-intervention establishes a minimally clinical important difference (MCID) when using the WOMAC as an evaluation tool.^[15]^ The study showed a greater than 11 point improvement (calculated mean difference between baseline and post-intervention testing) in both groups exposed to LLLT at T3 and T4. Functional improvements were observed by the increased number of timed sit-to-stand repetitions and improvements in knee flexion ROM. The findings from this study set up a discussion point for both structural and functional management traits in KOA. This research study suggests that analgesia associated with LLLT results in the anti-inflammatory properties on the articular capsule as suggested by the World Association of Laser Therapy (WALT), with similar results being produced, resulting in both pain relief and a decrease in knee circumference values.^[6,7,8]^

The main finding of this study is that of an adjunctive effect of LLLT with the conservative exercise management of KOA. The findings showed that the KOA outcomes significantly improved across all three intervention groups (p<0.05), with the LLLT group showing better short-term pain relief, but the combined exercise-LLLT group demonstrated better long-term reduction in pain symptoms. This implies a better residual effect when exercise treatment is applied with a laser. Indeed, fear of pain is the main reason for KOA patients reducing their physical activity.^[6]^ The combined exercise-LLLT treatment can improve both functional strength (short- and long-term), knee joint ROM and sit-to-stand scores thereby contributing to improved activities of daily living and quality of life. The findings of this study are supported by work undertaken by Alfredo et al. ^[6]^ where the benefits of combined intervention exposure were maintained for three months post exposure to the intervention.

In this present study participants were recruited from one geographic area and therefore further investigation is needed to confirm the findings in other geographic areas. Furthermore, participants were referred to the study by multiple medical practitioners and therefore a standard diagnostic criterion was not applied but it was rather a referral based on each practitioner’s specific diagnostic criteria.

## Conclusion

In conclusion, this 12-week RCT demonstrated that the addition of LLLT with exercise strengthens the effects on KOA outcomes, suggesting that the efficacy of LLLT as an adjunct form of therapy should be included in the non-surgical management of KOA. The data highlights the potential carryover effect of this tool in diagnosed KOA patients; however, further investigations are needed to confirm the observed effect.

## Figures and Tables

**Table 1:** Baseline and post-intervention characteristics

	Exercise (n=39)		LLLT (n=40)		Combined exercise-LLLT (n=32)	

	Baseline	Post-intervention	Baseline	Post-intervention	Baseline	Post-intervention
	
**Knee circumference Proximal patella (cm)**	41.4 ± 6.1	38.0 ± 6.1*	41.3 ± 6.1	40.0 ± 6.1*	43.5 ± 7.3	41.1 ± 7.4*
**Knee circumference Mid patella (cm)**	36.6 ± 5.1	38.9 ± 4.5*	38.5 ± 4.8	40.3 ± 4.5*	40.0 ± 4.6	39.2 ± 3.9*
**Knee circumference Distal patella (cm)**	37.4 ± 4.3	36.6 ± 4.2*	36.3 ± 4.7*	37.9 ± 3.1*	37.7 ± 4.6	37.5 ± 4.0*
**WOMAC**	56.6 ± 10.1	60.8 ± 9.8*	59.1 ± 10.2	61.5 ± 11.3*	56 ± 10.8	65.6 ± 9.9*
**ROM extension (** ⁰ **)**	2.1 ± 2.8	1.1 ± 1.9*	1.6 ± 2.5	1.4 ± 2.1*	1.4 ± 2.5	0.8 ± 1.6*
**ROM flexion (** ⁰ **)**	99.5 ± 14.6	102.3 ± 16.9	96 ± 17.4	103.7 ± 11*	95.2 ± 19.1	108.3 ± 11.9*
**Sit-to-stand (reps)**	17 ± 2.5	19.7 ± 3.5*	17.1 ± 2.9	19.6 ± 3.2*	17.4 ± 3.5	21.3 ± 4.1*

Data presented as mean ± SD.

* indicates p<0.05 vs. baseline values

LLLT, low-level laser therapy; WOMAC, Western Ontario and McMaster Universities Arthritis Index; ROM, range of motion;

⁰ , degrees; reps, completed repetitions.

**Table 2:** Temporal outcome measures for knee osteoarthritis (KOA) patients with different interventions

		Exercise (n=39)			LLLT (n=40)			Combined exercise-LLLT (n=32)		

Variable		T2	T3	T4	T2	T3	T4	T2	T3	T4
**Knee circumference Proximal patella (cm)**	**Mean ± SD**	40.0 ± 6.1*	38.3 ± 6.4*	38.0 ± 6.1*	38.0 ± 6.1*†	36.7 ± 6.1*†	35.2 ± 6.2*†	41.1 ± 7.4*	39.6 ± 6.7*†	38.3 ± 6.7*
	**Effect Size**	**0.2 (−0.2:0.7)**	**0.5 (−0.0:0.9)**	**0.6 (0.1:1.0)**	**0.3 (−0.1:0.8)**	**0.3 (−0.2:0.7)**	**0.4 (0.0:0.9)**	**0.2 (−0.6:0.3)**	**0.2 (−0.7:0.3)**	**0.1 (−0.5:0.4)**
**Knee circumference Mid patella (cm)**	**Mean ± SD**	40.3 ± 1.4	38.6 ± 4.5*	37.4 ± 4.5*	38.9 ± 4.5†	36.8 ± 4.2*†	36.0 ± 4.2*	39.2 ± 3.9†	37.0 ± 3.1*†	36.5 ± 3.7*†
	**Effect Size**	**0.1 (−0.6:0.3)**	**0.4 (−0.9:0.0)**	**0.2 (−0.6:0.3)**	**0.3 (−0.1:0.7)**	**0.4 (0.0:0.9)**	**0.3 (−0.1:0.8)**	**0.2 (−0.2:0.7)**	**0.4 (0.0:0.9)**	**0.2 (−0.2:0.7)**
**Knee circumference Distal patella (cm)**	**Mean ± SD**	37.9 ± 3.8*	38.0 ± 4.4	37.4 ± 3.6*	36.6 ± 4.2†	36.4 ± 4.2†	36.0 ± 4.2†	37.5 ± 4.0	37.3 ± 4.0	36.5 ± 3.7*†
	**Effect Size**	**0.1 (−0.6:0.3)**	**0.1 (−06:0.3)**	**0.0 (−0.5:0.4)**	**0.3 (−0.1:0.8)**	**0.4 (−0.1:0.8)**	**0.4 (−0.1:0.8)**	**0.1 (−0.4:0.6)**	**0.2 (−0.3:0.3)**	**0.3 (−0.2:0.7)**
**WOMAC**	**Mean ± SD**	61.5 ±11.3*	65.3 ±13.1*	70.5 ±11.8*	70.8 ± 9.8*†	76.9 ± 9.2*†	80.7 ± 8.5*†	65.6 ± 9.9*†	72.0 ± 8.7*†	78.0 ± 8.5*†
	**Effect Size**	**0.5 (0.9:0.0)**	**0.7 (−1.2:0.3)**	**1.3 (−1.7:0.8)**	**0.9 (−1.3:−0.4)**	**1.0 (−1.5:0.6)**	**1.0 (−1.4:−0.5)**	**0.4 (−0.8:0.1)**	**0.6 (−1.1:−0.1)**	**0.7 (−1.2:−0.2)**
**ROM extension (** ⁰ **)**	**Mean ± SD**	1.4 ± 2.1*	0.7 ± 1.5*	0.3 ± 0.9*	1.3 ± 1.9*	0.6 ± 1.3*	0.2 ± 0.6*	0.8 ± 1.6*†	0.4 ± 1.0*†	0.9 ± 0.4*†
	**Effect Size**	0.3 (−0.1:0.8)	0.6 (0.2:1.1)	0.9 (0.4:1.3)	0.1 (−0.4:0.5)	0.1 (−0.3:0.5)	0 (−0.4: 0.4)	0.3 (−0.2:0.8)	0.3 (−0.2:0.7)	0 (−0.5: 0.5)
**ROM flexion (** ⁰ **)**	**Mean ± SD**	103.7 ±13.4*	108.1 ± 12.4*	111.4 ± 11.5*	102.3 ± 16.9*	107.4 ± 14.9*	109.5 ± 13.2*	108.3 ± 11.9*†	112.3 ± 11.3*†	115.2 ± 10.3*†
	**Effect Size**	**0.3 (−0.7:0.2)**	**0.6 (−1.1:−0.2)**	**0.9 (−1.4:0.4)**	**0.1 (−0.4:0.5)**	**0.1 (−0.4:0.5)**	**0.2 (−0.3:0.6)**	**0.4 (−0.8:0.1)**	**0.4 (−0.8:0.1)**	**0.3 (−0.8:0.1)**
**Sit-to-stand (reps)**	**Mean ± SD**	19.5 ± 3.0*	23 ± 4.5*	26 ± 3.5*	19.7 ± 3.5*	25.5 ± 3.0*†	25.5 ± 4.0*	21.2 ± 4.0*†	25.5 ± 3.0*†	30.0 ± 3.0*†
	**Effect Size**	**0.9 (−1.4:−0.4)**	**1.6 (2.1:−1.1)**	**0.3 (−3.6:−2.3)**	**0.1 (−0.5:0.4)**	**0.7 (−1.1:−0.2)**	**0.1 (−0.3:0.6)**	**0.5 (−0.9:0.0)**	**0.7 (−1.1:−0.2)**	**1.2 (−1.7:−0.7)**

Data presented as mean ± SD with effect sizes (95% CI). Bold font indicates effect size >0.02;

* indicates p<0.05 (T1 vs T2, T1 vs. T3, T1 vs. T4) using independent tests;

† indicates p<0.05 exercise vs. LLLT, and exercise vs. combined exercise-LLLT.

LLLT, low-level laser therapy; WOMAC, Western Ontario and McMaster Universities Arthritis Index; ROM, range of motion;

⁰ , degrees; reps, completed repetitions; T2, post intervention; T3, 1 month post-intervention; T4, 3 months post intervention.
